# Role of Surface Topography in the Superhydrophobic Effect—Experimental and Numerical Studies

**DOI:** 10.3390/ma15093112

**Published:** 2022-04-25

**Authors:** Samih Haj Ibrahim, Tomasz Wejrzanowski, Bartłomiej Przybyszewski, Rafał Kozera, Xabier García-Casas, Angel Barranco

**Affiliations:** 1 Faculty of Materials Science and Engineering, Warsaw University of Technology, Woloska 141, 02-507 Warsaw, Poland; tomasz.wejrzanowski@pw.edu.pl (T.W.); bartlomiej.przybyszewski.dokt@pw.edu.pl (B.P.); rafal.kozera@pw.edu.pl (R.K.); 2 Technology Partners Foundation, Pawinskiego 5A, 02-106 Warsaw, Poland; 3 Nanotechnology on Surfaces and Plasma Group (CSIC-US), Materials Science Institute of Seville (Consejo Superior de Investigaciones Científicas—Universidad de Sevilla), c/Américo Vespucio 49, 41092 Seville, Spain; xabier.garcia@icmse.csic.es (X.G.-C.); angel.barranco@csic.es (A.B.)

**Keywords:** superhydrophobic surfaces, roll-off angle, wettability

## Abstract

Within these studies, the effect of surface topography for hydrophobic coatings was studied both numerically and experimentally. Chemically modified polyurethane coating was patterned by application of a laser beam. A set of patterns with variously distant linear peaks and grooves was obtained. The cross section of the pattern showed that the edges of the peaks and grooves were not sharp, instead forming a rounded, rectangle-like shape. For such surfaces, experimental studies were performed, and in particular the static contact angle (SCA), contact angle hysteresis (CAH), and roll-off angle (ROA) were measured. Profilometry was used to create a numerical representation of the surface. Finite volume method was then applied to simulate the behavior of the water droplets. The model developed herewith enabled us to reproduce the experimental results with good accuracy. Based on the verified model, the calculation was extended to study the behavior of the water droplet on the simulated patterns, both spiked and rectangular. These two cases, despite a similar SCA of the water droplet, have shown extremely different ROA. Thus, more detailed studies were dedicated to other geometrical features of such topography, such as the size and distance of the surface elements. Based on the results obtained herewith, the future design of superhydrophobic and/or icephobic topography is discussed.

## 1. Introduction

The hydrophobic effect has recently garnered much wider research interest due to an increased number of applications. Self-cleaning surfaces, rain-repellant windows, anti-biofouling ship hulls, corrosion resistant coatings, and anti-icing surfaces are only a section of potential applications [1]. Hence, an understanding of wetting phenomena is necessary for further development of these technologies.

Wetting of surfaces can be divided into two regimes: hydrophilic, with a contact angle (CA) of less than 90°, and hydrophobic, with higher contact angles [2]. The CA of the liquid depends on the surface energy of the droplet, in accordance with the Young–Dupre equation [3]:

(1) $$\cos\theta =\frac{\gamma_{SV}-\gamma_{SL}}{\gamma_{LV}}$$

where *γ_SV_*, *γ_SL_*, and *γ_LV_* are surface tensions for interfaces between solid *S*, liquid *L*, and vapor *V*, respectively. This equation is valid only for perfectly flat surfaces. When the real topography of the solid substrate surface is included in Equation (1), the wetting phenomenon becomes much more complex. Depending on the chemistry and roughness of the surface, wetting can be divided into two main regimes: (1) homogeneous wetting (Wenzel model) and (2) heterogeneous wetting (Cassie–Baxter model) [4]. Homogeneous wetting can be described with the Wenzel equation:

(2) $$\cos\theta_{w}=r\frac{\gamma_{SV}-\gamma_{SL}}{\gamma_{LV}}=r\cos\theta$$

where *r* is a roughness factor defined as the ratio of the true area of a rough surface to the projected area, and $\theta_{w}$ is the apparent contact angle. In this model, liquid infiltrates the grooves of the rough surface, therefore it is a good representation of hydrophilic wetting phenomenon [5].

Heterogeneous wetting is based on the assumption of two different materials participating in the wetting phenomenon [6]. By applying the mixture rule, the effect of each phase on resultant contact angle can be described with the equation:

(3) $$\cos\theta =f_{1}\cos\theta_{1}+f_{2}\cos\theta_{2}$$

where *f*_1_, *f*_2_ are surface area fractions and $\theta_{1}$, $\theta_{2}$ are contact angles on phase 1 and 2. Hence, it is possible to consider air bubbles trapped in hollows on the surface as a second phase, simply by assuming that the air phase has a contact angle of 180°. Therefore, the Cassie–Baxter equation with surface roughness considered can be written as follows [7]:

(4) $$\cos\theta_{CB}=rf\cos\theta_{1}+\left(1-f\right)\cos180{}^{\circ}=rf\cos\theta_{1}+f-1$$

Therefore, it can be applied to model hydrophobic wetting. As the abovementioned equations suggest, the contact angle of the surface can be modified in two ways, either by changing the surface tension of the liquid–solid interface (i.e., by changing the chemical composition of the surface) or by changing the roughness factor (the topography of the surface) [8].

Excellent hydrophobic property (CA > 150°) accompanied with low roll-off angle combine to create so-called superhydrophobic properties. It can be achieved by simultaneous modification of the surface chemistry and topography. Changing only the surface chemistry is not enough to achieve superhydrophobicity. Examples can be found in fluorine polymers, which exhibit high water repellency by means of reducing surface free energy, which is the quantity of molecular bond disruption due to the creation of free surface. Surfaces with a lower value of surface free energy tend to have a higher liquid repellency. Regularly aligned closest-hexagonal-packed trifluoromethyl groups, as a material with the lowest value of surface free energy, achieves only a 120° static contact angle [9]. One of the wetting models, i.e., Cassie–Baxter for textured surfaces with different scales of roughness, explains the possibility for obtaining a significant increase in water repellence [10,11]. Such wetting properties can be described by three basic parameters: static contact angle (SCA), contact angle hysteresis (CAH), and roll-off angle (RoA). According to the definition, the solid surface can be classified as superhydrophobic when CA is higher than 150°, and CAH and RoA are less than 10°. Many authors [12,13,14,15] showed that surfaces fulfilling the Cassie–Baxter wetting state displayed increased water repellency, improvement of self-cleaning properties as well as reduction of surface corrosion and decrease in ice adhesion that is often associated with smaller CA hysteresis which is typical for a surface exhibiting the Cassie–Baxter wetting model. It can be explained by the relatively small liquid–solid interface contact area, which encourages water droplet roll-off [16,17].

There are many methodologies for obtaining the superhydrophobic properties of a surface. One of them is texturization to hierarchical micro-and nano-scale patterns with a combination of chemical treatments in volume materials [18] to obtain low surface energy, which in turn favors icephobic properties. One of the methodologies utilized to achieve roughness for hydrophobic surfaces is laser patterning. Depending on the wavelength and duration of the pulse beam, the contact angle can be tailored to achieve high values [19]. There are also several other methods for surface texturization. Most popular are lithography [20], plasma etching [21], chemical mask etching [22] and laser patterning [23,24].

When comparing the performance of the two scales, i.e., micron and nanoscale roughness, the latter possibly possesses better hydrophobic and icephobic behavior [25,26,27,28]. Another example is that for fractal surfaces, exhibiting roughness in all size scales, reported contact angles can achieve values as high as 174° [29]. Moreover, the presence of air pockets, formed in the grooves of the surface at the solid/liquid interface when contacting water droplets, is favorable for obtaining a higher water repelling ability. However, Varanasi et al. [30] have found that frost formation inside the textures of superhydrophobic surfaces in turn could increase the ice adhesion.

Many papers have indicated no direct relationship between hydrophobicity and icephobicity, especially when the water/substrate contact angle is considered. There is a strong interest from the scientific community in determining appropriate surface parameters and properties for superhydrophobic surfaces as well as techniques for their examination. The most dominant theory is that superhydrophobic surface with high water repellence exhibit improved anti-icing properties. Due to their potent ability to delay and/or reduce ice accumulation, snow, and frost, they are reported as coatings with high ice phobic potential [31,32,33,34,35,36]. Among many publications, several authors highlighted the effect of contact angle hysteresis on the ice adhesion strength [18,31,32,33,36], while others have reported reduced ice adhesion for high water contact angle values [31,32,33,34,35]. In opposition to those findings, many authors indicate no correlation or even negative influence of superhydrophobic properties on icephobic behavior. For example, recently, Chen et al. [28] suggested that superhydrophobic properties cannot decrease the ice adhesion value, while those parameters were almost constants independently of the hydrophobicity values. A similar relationship was partly found by Kulinich et al. [31] for rough hydrophobic surfaces, with the exception that wetting hysteresis was well corelated with ice adhesion, while Meuler et al. found a strong correlation for silicon-based materials, reserving that it is true more for rigid materials than for elastomeric substrates [25]. Many authors have shown that high contact angle combined with low roll-off angle results in a superhydrophobic and also icephobic material [37,38]. Thus, understanding the effect of the surface topography, not only on the contact angle but also on other dynamic parameters, such as roll-off angle, becomes important.

The next factor which is studied by several authors is the presence of polar components on the surface, which reduces adhesion between the water droplet and surface due to weaker hydrogen bonds, which in turn facilitate water droplets rolling off and supports the minimalization of icing accretion [30,39].

In nature, the superhydrophobic effect is common, and it can be observed in, e.g., leaves, feathers, or aquatic insects. The most common example is the lotus effect, coming from lotus leaves, which exhibit superior superhydrophobic and self-cleaning properties [40]. Only after the development of the scanning electron microscope, which rendered it possible to observe fine details of the surface microstructure, was this finding explained. Macroscopically smooth surfaces observed with higher magnification exhibited hierarchical roughness in the micro- and nanoscale [41]. This structural feature coupled with hydrophobic epicuticular wax crystalloids on the surface is the cause of the excellent superhydrophobic effect.

However, in some cases, the geometry of the surface can inhibit the droplet sliding. A fine example is the rose petal effect, in which a droplet is stuck on the petal, despite the very high contact angle of the surface [42]. Hence, study of the influence of surface roughness on water adhesion/sliding on the surface is necessary to understand the superhydrophobic effect. Miwa et al. studied the effect of surface roughness and contact angles on the sliding of the droplet [43]. They derived the relation between sliding and contact angles:

(5) sinα=2rk sinθ′(cosθ′+1)g(rcosθ+1 ){3π2m2ρ(2−3cosθ′+cos3θ′)}13

where *r* is the roughness factor, θ is the contact angle, $\theta^{\prime }$ is the apparent contact angle, *m* is the mass of the droplet, ρ is the density of water and *k* is the proportionality constant.

However, this analytical model does not explain the mechanism of the droplet sliding. Thus, modeling research is necessary to explain the droplet sliding phenomenon and the complex hydrodynamics of wetting. Computational fluid dynamics (CFD) was utilized in a vast number of studies on droplet dynamics and their interaction with solid surfaces. Volume of fluid method (VOF) is the main technique used to track the free-surface (i.e., interface between air and water) of the droplet [44,45,46,47]. In this fixed-grid method, the interface is modeled as the discontinuity in volume fraction function. It can be utilized successfully to develop water droplet models and simulations of droplet spreading on various surfaces [45,48]. It can be also utilized to simulate droplet collision on a flat surface and subsequent spread/recoil [49,50]. It was even proven to work for more complex simulations of droplet behavior on flat surfaces i.e., droplet freezing [51], just by the addition of modules (heat transfer equations, solidification/melting etc.). However, those approaches did not include the roughness of the surface. Chamakos et al. [52] tried to tackle this problem by changing the boundary condition to account for surface roughness, but this approach only averages the fine details of the surface topography, which is not applicable for more complex surface patterns.

In this paper, a novel CFD model is developed to study the effect of local surface roughness on droplet spreading and sliding behavior. Numerical analysis was performed using topography of real samples with their surfaces patterned by laser beam. The samples were also investigated experimentally, enabling model verification. Afterwards, new artificial patterns were studied to determine the influence of shape and geometry on droplet sliding behavior.

## 2. Materials and Methods

A 3-component PUR topcoat (Aviox^®^ finish 77702) was obtained from Akzo Nobel, The Netherlands. It was used in combination with the Aerodur^®^ HS 37092 2-component amine-cured epoxy primer (Akzo Nobel, Sassenheim, The Netherlands). To modify the topcoat, Fluorosil^®^ OH C7-F (Siltech, Toronto, ON, Canada), a liquid fluoroalkyl and alkylcarbinol functionalized silicone based on non-PFOS fluoroalkyl chains was utilized. Silicone-based additives are promising for modification of polyurethanes due to the higher energy of bonds (Si-O-Si, 550 kJ·mol^−1^) and (Si-C, 369 kJ·mol^−1^) present in the silicone chain, in comparison to the energy bonds (C-C, 347 kJ·mol^−1^), (C-O, 340 kJ·mol^−1^) and (N-C, 335 kJ·mol^−1^) existing in polyurethane. Hydrophobicity of the alkyl groups present in the Fluorosil additive causes increased interfacial energy between silicone and polyurethane matrix. A decrease in interfacial energy is achieved via the migration of silicon to the coating surface. This results in altered surface properties including increased hydrophobicity and chemical resistance, even at a low silicon content. [53]. The hydrophobic nanosilica Aerosil^®^ R805 (Evonik, Hanau, Germany) used in this study has a specific surface area of 125–175 m^2^·g^−1^ and a nominal particle size of 15 nm. It was produced by treating nano-silica with organosilane. The aim of the modified nanosilica addition was to roughen the surface with low surface energy materials and to achieve dual-scale (micro and nano) hierarchical structure. This is one of the essential factors in generating hydrophobic coatings and especially important for obtaining surfaces with low water sliding angles [54]. All the materials mentioned above were used as received. The modified PUR coatings were deposited on 2024 aluminum alloy (WMH Group, Essen, Germany).

The 2024 CLAD aluminum alloy coupons were first cleaned in an ultrasonic acetone bath for 15 min. Initially, both primer components were mixed according to the manufacturer’s indications, then sprayed on Al coupons by means of a Walther^®^ Pilot XIII air spray gun (Wuppertal Vohwinkel, Germany) and left to dry for 3 h. Meanwhile, PUR topcoats with 3 wt.% of nanosilica and Fluorosil additive (5 wt.%) were prepared by mixing using a magnetic stirrer. Before spraying, nano-silica suspensions on the modified PUR coating were prepared by means of an ultrasonic gun (VCX 750, Sonics and Materials Inc., Newtown, CT, USA) for 30 min. The suspension was then sprayed on top of the already applied primer and left to dry for 24 h.

To achieve superhydrophobic properties and decrease the roll-off angle, coupons coated with hybrid-modified polyurethane were subjected to UV laser patterning. The samples were processed in a laser marking platform controlled by CAD using a fixed optical head equipped with a 50× processing objective. The samples were mounted in a two-axis computer-controlled motorized stage holder with a XY repeatability of 400 nm. The laser source was a 300 mW, 355 nm diode pumped solid state q-switch laser Explorer One from Spectra-Physics. The laser was operated at 10% of the total power without attenuators. Laser cut speed was 1 mm·s^−1^ and the jump speed was 10 mm·s^−1^. The laser spot width was 5µm, so it was possible to obtain overlapping lines for displacement values lower than the beam width. The fabricated surfaces had different patterns and geometry; nine surfaces of parallel laser beam lines with different width and distances between each other were produced. The depth of the grooves was 8 µm. All combinations of groove width and peak width from 5, 10 and 20 µm were manufactured, which resulted in 9 different surface patterns. Samples were denoted as X by Y, where X is peak width and Y is groove width. For example, sample 20by10 has a surface pattern with 20 µm peak width and 10 µm groove width.

**Figure 1:**
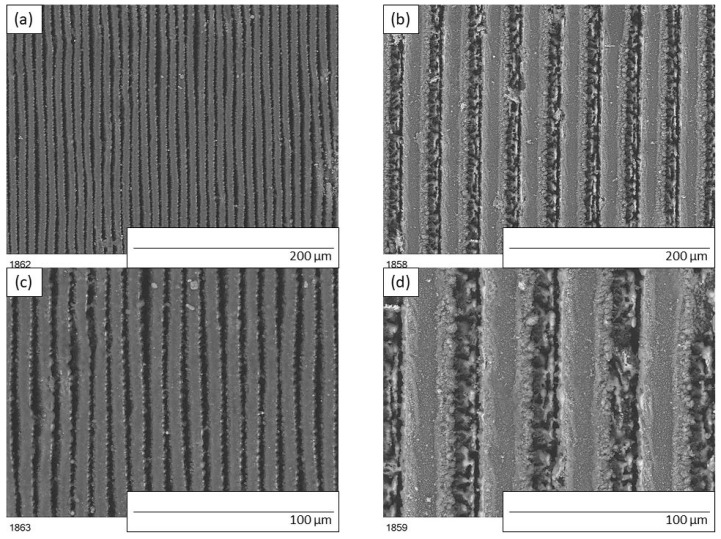
SEM images of modified polyurethane coatings after laser patterning: (**a**,**c**) sample ‘5by5’, (**b**,**d**) sample ‘20by20’.

The geometry of the patterns assumed before laser patterning were validated by scanning electron microscopy. Peak and groove width were measured and found to be slightly different than designed. In Figure 1a top view of samples ‘5by5’ and ‘20by20’ are presented.

Surface profiles were measured by means of a contact profilometer (SJ-310, provided by Mitutoyo, Kanagawa, Japan). All measurements were acquired from 10 mm segments. Six different, randomly selected profiles were measured on each modified sample.

Wettability properties (static contact angle (SCA), advancing contact angle (ACA), receding contact angle (RCA), roll-off angle (RoA)) were measured by a contact angle measurement system (Data Physics GmbH OCA 15, Filderstadt, Germany). All angles of each sample were measured at least three times across the sample surface using the sessile drop method, by dispensing 3 µL (SCA), 5 µL (ACA, RCA) and 10 µL (RoA) of deionized water on the sample’s surface. The contact angle hysteresis (CAH) was calculated as the difference between ACA and RCA.

The microstructure of the coating surfaces was observed with a scanning electron microscope (SEM, Hitachi S5500, Tokyo, Japan) operated at 5–15 kV. All samples were sputtered with a gold-palladium coating.

To simulate a droplet rolling off from a rough surface, a 2D simulation domain (5 mm width and 3 mm height) of area above the material surface was generated in Ansys SpaceClaim. The lower boundary representing the material surface was generated on the basis of profilometry results, as a set of lines connecting points obtained during the surface topography testing. Such an approach enabled us to verify the results of the numerical simulations by comparison with experimental roll-off-angle measurements.

Additionally, models with artificial surface topography were generated to study the influence of different shapes and distances between grooves on the wetting phenomenon. The pattern of micrometric grooves was translated over 200 times and the resulting geometry was big enough to simulate the behavior of a droplet in millimeter scale without losing fine details of surface roughness.

**Figure 2:**
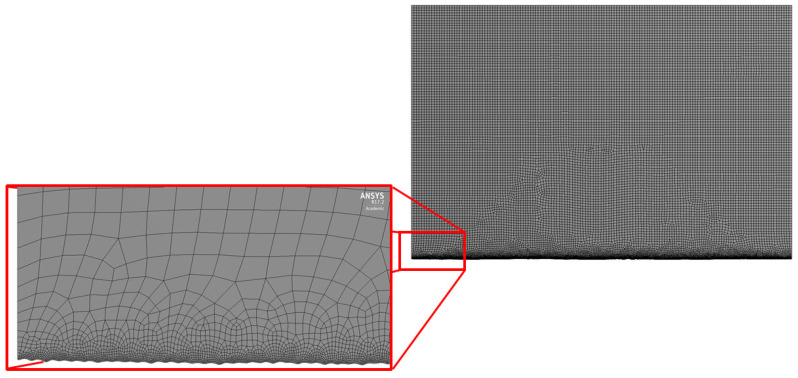
Example of the generated mesh for further calculations.

Generated 2D geometry was meshed in Ansys Design Modeler. Named selections of model edges were specified for further specification of boundary conditions during the simulation set-up. The global maximum element size was set to 20 µm with a refinement of size 1 µm on the lower boundary representing the material surface. Quad type elements were mostly present, with the exception of some triangular elements in the mesh size transition zone. The final number of elements for each model was around 100,000 for such settings. In Figure 2, an example of the meshed geometry is presented.

Simulation of the roll-off angle phenomena was performed in Ansys Fluent. Volume of fluid (VOF) method was used as a multiphase interface tracking technique. Gravitational acceleration was set to Y-direction with a standard value of 9.81 m·s^−2^. Air and liquid water properties were imported from the Fluent database. Water surface tension was set to a value of 0.072 N·m^−1^ with the continuum surface force model. The wall adhesion option was enabled, so that the contact angle with the material surface could be set. Implicit body force correction in the momentum equation was included, due to the existence of large body forces (gravity, surface tension force) in the studied phenomenon. A standard operating condition of 1013.25 hPa pressure and 1.225 kg·m^−3^ air density were set. The following boundary conditions were set:

- Velocity inlet with 0 m·s^−1^ velocity magnitude on the upper edge
- Outflow on the sides of simulation domain
- Wall with No Slip condition and static contact angle on the lower boundary representing the material’s surface.

**Figure 3:**
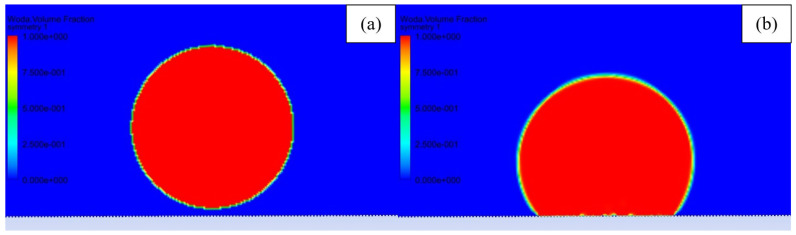
Volume fraction of fluid (red—water, blue—air) presenting: (**a**) Initial droplet configuration and (**b**) droplet after stabilization.

The fractional step scheme of pressure velocity coupling was chosen to improve the speed of calculation. The non-iterative time-advancement scheme of transport equation solving was enabled to speed up the transient simulation. Improving the speed of calculation was necessary because a very small element size induced a very small timestep of 2 × 10^−7^ s, due to Courant number limitation. The simulation was initialized with initial values of 0 Pa pressure and 0 m·s^−1^ velocity. A circle with a radius of 1.06 mm located 0.05 mm above the material’s surface was patched with a volume fraction of 100% water, and rest of the simulation domain was patched with air. Such a radius was picked to achieve 5µL volume, similar to the droplet size in experimental testing. The droplet was set on the surface after calculating 100,000 timesteps of the droplet falling on the surface and stabilization. In Figure 3, the initial configuration of the simulation was presented. Simulation was performed with 5° mesh rotation after 100,000 timestep intervals. The roll-off angle was determined when the droplet shifted more than the distance of 2 grooves from the initial position. The 2 grooves shift condition for evaluating roll-off was picked after a series of modeling for the samples, which exhibited high droplet adhesion. In those cases, the droplet did not roll off after 90° rotation, and it did not move even one groove distance.

**Figure 4:**
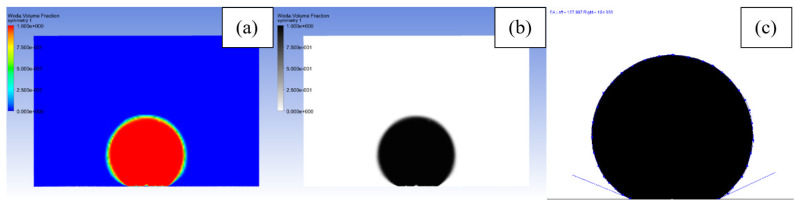
Measurement of contact angle: (**a**) initial volume fraction image (red—water, blue—air), (**b**) grayscale volume fraction image (black—water, white—air), and (**c**) contact angle measured with drop analysis plugin.

Apparent contact angle was measured after droplet stabilization. In the first step, the grayscale image with water volume fraction in the simulation domain was exported from Ansys CFD Post software. This image was loaded in ImageJ software, cropped to the right size, and binarized. Afterwards, the contact angle was determined using the DropAnalysis plugin [55], dedicated to droplet contact angle measurements. It is based on B-Spline Snake Approach, in which active contours are used to shape the droplet. The whole process of preparation for contact angle measurement was illustrated in Figure 4.

## 3. Results and Discussion

Surface wettability of the modified polyurethane coatings after laser patterning was investigated by water contact angle measurements. SCA, CAH, and RoA values for all manufactured samples are shown in Table 1.

Polyurethanes are hydrophilic polymers due to the polar groups present in their chain structure. In fact, the SCA, CAH, and RoA of the reference coating were 85°, 39°, and 87°, respectively, confirming the non-hydrophobic behavior of this material. The use of nanosilica and silicone-based modifiers caused an improvement in hydrophobic properties due to the differences in roughness and surface free energy. Despite this, superhydrophobic behavior was still not achieved (SCA, CAH, and RoA were 107°, 35° and 80°, respectively). UV laser patterning caused a significant increase in hydrophobic properties. For the samples with a groove width of 20 µm (20by20, 5by20, 10by20), the measured static contact angle was above 130°. The greatest hydrophobic properties were observed for sample ‘20by20’, where SCA was 136° and CAH and RoA were 5°.

**Figure 5:**
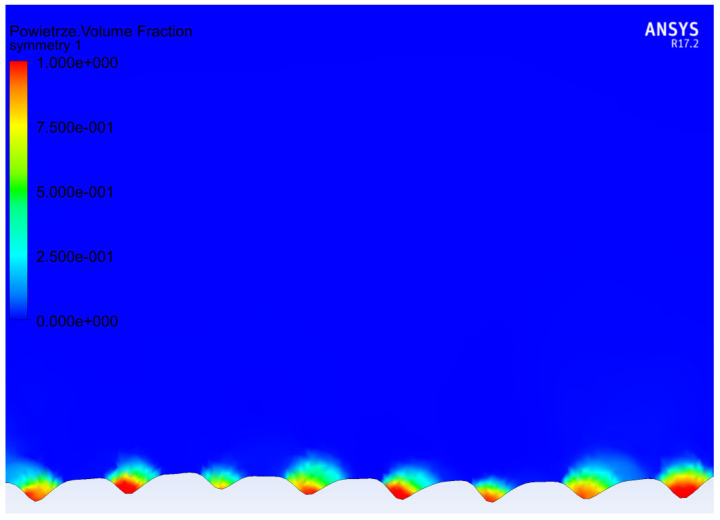
Volume fraction of the fluid in the grooves: blue—water and red—air. Visible air pockets trapped in the grooves.

To validate the numerical calculations, the surface profiles of the samples, for which the contact angle and roll-off angle were tested, were converted into finite volume meshes, and roll-off angles from sliding simulations were compared with the experimental results. In Table 2, the results of the comparison are presented. For all samples, the apparent contact angle determined by numerical simulations was smaller than the experimental result by around 5°. For samples 5by5 and 10by10, the droplet did not roll off the surface, even with 90° surface tilt. That happened in both experimental tests and numerical simulations. Similarly, by utilizing the analytical model proposed by Miwa et al. [43] (formula nr 5), the sinus of the sliding angle is greater than 1 for these samples, which does not provide a valid result. In the case of 20by20 samples, the experiment indicated an excellent roll-off angle of 5°. However, in numerical simulations the roll-off angle has a value of 30°. The value of the surface contact angle for the flat surface may have changed due to laser treatment. Hence, additional simulations with higher contact angle on the material surface were performed. For a base contact angle equal to 140°, the apparent contact angle and roll-off angle values were much closer to the values determined experimentally. As can be noticed, the numerical simulations provided a much closer outcome than the analytical model provided by Miwa [43]. This may be caused by some of the model’s assumptions not being fulfilled. In Figure 5, the volume fraction of air in the grooves is presented for one of the models. Visible air pockets suggest the Cassie–Baxter model of wetting. Similar air pockets were visible in all models.

**Figure 6:**
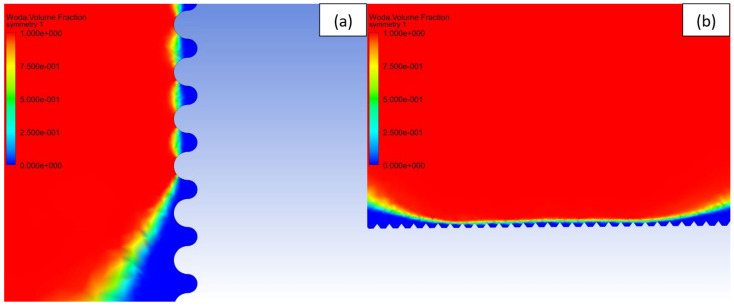
Volume fraction of the fluid in the grooves: blue—air and red—water. (**a**) Water phase does not reach the next bulge on circular pattern, (**b**) Water-air interface with the spiked pattern.

To study the effect of the shape of the surface pattern on the hydrophobic properties, 5 different patterns with similar intervals of 20 μm were generated (see Table 3) and sliding simulations were performed with 120° contact angle conditions. As can be noticed, despite a slight increase in the apparent contact angle, the droplet did not roll off from the rectangular and circular patterns. A close-up on the front of the droplet (see Figure 6a) revealed that the water phase sticks to the edge of the bulge and the droplet does not move forward. This suggests that surfaces meant to be superhydrophobic should have a net-like pattern rather than columnar, so the water phase could propagate on the surface. Otherwise, the droplet sliding behavior will be similar to the rose petal effect. However, in the case of spiked patterns, very high apparent contact angles and low roll-off angles were obtained. This may be caused by the very small contact area between the material surface and the droplet (see Figure 6b). Similar effects can be found in lotus leaves with pointy protrusions.

To study the effect of the spacing between bulges of the surface pattern on the hydrophobic properties, two sets of surface patterns with increasing groove width were generated: rectangular and spiked. In Table 4, the results of simulations of the droplet sliding on surfaces with rectangular pattern are presented. It can be found that the increase in the groove width from 5 µm to 20 µm did not change the droplet behavior—it did not roll off from the surface, even with 90° surface tilt. However, a slight increase in the apparent contact angle was observed, from 145° for the 5by5 μm patterns to 149° for the 20by5 μm patterns. It can be noticed that the shape of the surface pattern may have much bigger impact on the sliding behavior of the droplet.

In Table 5, the results from simulations for the spiked patterns are presented. High apparent contact angle values were obtained (~170°). However, no clear relationships between pattern spacing and apparent contact angle were observed. The increase in the groove width resulted in a decrease in the roll-off-angle, from 15° for 5 μm distance between bulges to 5° for 20 μm distance. However, that relationship should be true only in the Cassie–Baxter wetting regime. Further increase in the pattern spacing may cause the water phase to infiltrate the grooves. Thus, the spacing between spikes in such a pattern needs to be fine-tuned to the wetting parameters (i.e., contact angle) of the material surface to achieve superhydrophobic properties.

## 4. Conclusions

Both the chemical composition and topography of the surface influence the wetting phenomenon when the superhydrophobic effect is considered. The literature review indicates that hydrophobic surfaces with an SCA of 100–120 degree can be obtained by chemical modification only (even for very flat surfaces). However, to achieve an SCA over 120 degrees, the specific topography must be shaped additionally.

Within these studies, a numerical model has been developed, enabling the simulation of water droplet behavior on the surface with defined topography. In the first stage, the model was verified experimentally by using the topography data from profilometry of the surface treated by a laser beam. Later, the model was applied to study idealized cases enabling to identify geometrical features of the topography crucial for water and ice interaction with the materials surface.

The results show that the shape of peaks is the most important feature of the surface topography. For the rectangular-like shape, the droplet sticks to the surface even with a tilt angle of 90 degrees. Changing the distance between peaks from 5 to 20 µm did not change this effect. Different behavior of the water droplet was been observed for spike-like topography. In this case, increasing the distance between peaks from 5 to 20 µm reduced the ROA from 15 to 5 degrees, respectively.

Since these studies have a preliminary character, they demonstrate the methodology and trends in the design of topography for superhydrophobic surfaces. It also confirms that SCA or ACA are not sufficient to define the superhydrophobic nature of the surface. It seems that ROA better describes this effect. However, a more extensive analysis is planned to establish more quantitative relationships between surface topography and wetting parameters.

## Figures and Tables

**Table 1:** Wettability parameters of polyurethane coatings after laser patterning.

Sample	SCA [°]	CAH [°]	RoA [°]
Reference PUR	85	39	87
Modified PUR	107	35	80
5by5	114	18	90
10by5	119	20	45
20by5	115	25	90
5by10	118	23	40
10by10	121	26	90
20by20	136	5	5
5by20	133	24	40
10by20	131	18	35
20by10	115	27	70

**Table 2:** Comparison of modeling and experimental results of roll-off angles for different samples.

Sample	Experiment		Model		Model (140° Surface Contact Angle)		Model Miwa et al. [43]	
	Apparent Contact Angle [°]	Roll-off-Angle [°]	Apparent Contact Angle [°]	Roll-off-Angle [°]	Apparent Contact Angle [°]	Roll-off -Angle [°]	107 CA [°]	140 CA [°]
5by5	114	>90	107	>90	113	>90	-	-
10by10	121	>90	116	>90	122	>90	-	-
20by20	136	5	132	30	138	10	66	27

**Table 3:** Surface patterns for modeling studies and results of ROA and ACA.

Surface Pattern		Roll-Off Angle [°]	Apparent Contact Angle [°]
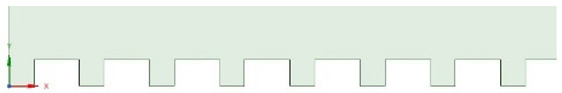	Rectangle	>90	128
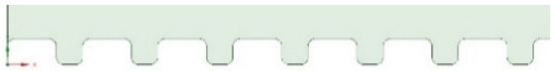	Rounded rectangle	>90	133
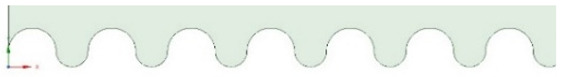	Circles	>90	130
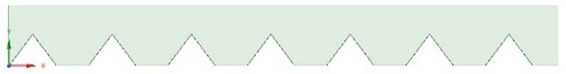	Spikes	10	167
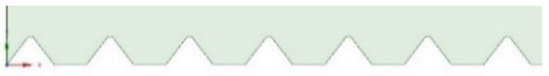	Rounded spikes	35	166

**Table 4:** Results of simulation of droplet sliding on surfaces with rectangular pattern.

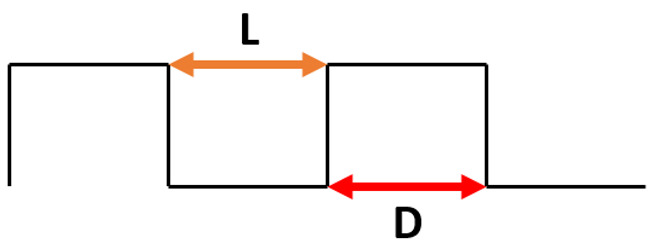	**Surface Pattern** **L_D [µm]**	**Roll-Off Angle** **[°]**	**Apparent Contact Angle** **[°]**
	5_5	>90	145
	10_5	>90	146
	15_5	>90	147
	20_5	>90	149

**Table 5:** Results of simulation of droplet sliding on surfaces with spiked pattern.

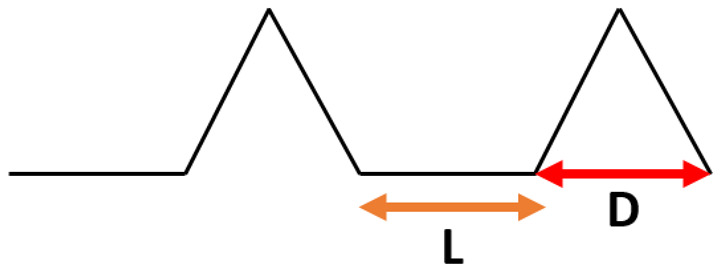	**Surface Pattern L_D [µm]**	**Roll-Off Angle [°]**	**Apparent Contact Angle [°]**
	5_12	15	172
	10_12	10	169
	15_12	10	175
	20_12	5	170

## Data Availability

The data presented in this study are available on request from the corresponding author. The data are not publicly available due to ongoing research in the project.

## References

[1] Li X.M., Reinhoudt D., Crego-Calama M. (2007). What do we need for a superhydrophobic surface? A review on the recent progress in the preparation of superhydrophobic surfaces. Chem. Soc. Rev..

[2] Vinogradova O.I., Belyaev A.V. (2011). Wetting, roughness and flow boundary conditions. J. Phys. Condens. Matter.

[3] Young T. (1805). An essay on the cohesion of fluids. Philos. Trans. R. Soc. London.

[4] Grewal H.S., Cho I.J., Oh J.E., Yoon E.S. (2014). Effect of topography on the wetting of nanoscale patterns: Experimental and modeling studies. Nanoscale.

[5] Wolansky G., Marmur A. (1999). Apparent contact angles on rough surfaces: The Wenzel equation revisited. Colloids Surfaces A Physicochem. Eng. Asp..

[6] Cassie A.B.D. (1948). Contact angles. Discuss. Faraday Soc..

[7] Marmur A. (2004). The lotus effect: Superhydrophobicity and metastability. Langmuir.

[8] Guo Z., Liu W., Su B.L. (2011). Superhydrophobic surfaces: From natural to biomimetic to functional. J. Colloid Interface Sci..

[9] Nishino T., Meguro M., Nakamae K., Matsushita M., Ueda Y. (1999). The lowest surface free energy based on -CF3 alignment. Langmuir.

[10] Cassie A.B.D., Baxter S. (1944). Wettability of porous surfaces. Trans. Faraday Soc..

[11] Gregorčič P., Conradi M., Hribar L., Hočevar M. (2018). Long-term influence of laser-processing parameters on (Super)hydrophobicity development and stability of stainless-steel surfaces. Materials.

[12] Kreder M.J., Alvarenga J., Kim P., Aizenberg J. (2016). Design of anti-icing surfaces: Smooth, textured or slippery?. Nat. Rev. Mater..

[13] Shirtcliffe N.J., McHale G., Atherton S., Newton M.I. (2010). An introduction to superhydrophobicity. Adv. Colloid Interface Sci..

[14] Yeong Y.H., Milionis A., Loth E., Sokhey J., Lambourne A. (2015). Atmospheric Ice Adhesion on Water-Repellent Coatings: Wetting and Surface Topology Effects. Langmuir.

[15] Barrios J.M., Romero P.E. (2019). Improvement of surface roughness and hydrophobicity in PETG parts manufactured via fused deposition modeling (FDM): An application in 3D printed self-cleaning parts. Materials.

[16] Giacomello A., Meloni S., Chinappi M., Casciola C.M. (2012). Cassie-baxter and wenzel states on a nanostructured surface: Phase diagram, metastabilities, and transition mechanism by atomistic free energy calculations. Langmuir.

[17] Bhushan B., Nosonovsky M. (2010). The rose petal effect and the modes of superhydrophobicity. Philos. Trans. R. Soc. A Math. Phys. Eng. Sci..

[18] Kozera R., Przybyszewski B., Krawczyk Z.D., Boczkowska A., Sztorch B., Przekop R.E., Barbucha R., Tański M., Casas X.G., Borras A. (2020). Hydrophobic and anti-icing behavior of uv-laser-treated polyester resin-based gelcoats. Processes.

[19] Irzmańska E., Korzeniewska E., Pawlak R., Tomczyk M., Smejda-Krzewicka A., Adamus-Włodarczyk A. (2022). Enhanced Hydrofobicity of Polymers for Personal Protective Equipment Achieved by Chemical and Physical Modification. Materials.

[20] Bhushan B., Her E.K. (2010). Fabrication of Superhydrophobic Surfaces with High and Low Adhesion Inspired from Rose Petal. Langmuir.

[21] Somrang W., Denchitcharoen S., Eiamchai P., Horprathum M., Chananonnawathorn C. (2018). Superhydrophobic and antireflective surface of nanostructures fabricated by CF4 plasma etching. Mater. Today Proc..

[22] Leese H., Bhurtun V., Lee K.P., Mattia D. (2013). Wetting behaviour of hydrophilic and hydrophobic nanostructured porous anodic alumina. Colloids Surf. A Physicochem. Eng. Asp..

[23] He H., Wu W., Xi Z., Ma Z., Zhang L., Wang C., Sun L. (2022). Time dependency of superhydrophilic and superhydrophobic surfaces produced by nanosecond laser irradiation assisted by post-annealing and silanization. Appl. Surf. Sci..

[24] Gao J., Wu Y., Zhang Z., Zhao D., Zhu H., Xu K., Liu Y. (2022). Achieving amorphous micro-nano superhydrophobic structures on quartz glass with a PTFE coating by laser back ablation. Opt. Laser Technol..

[25] Meuler A.J., Smith J.D., Varanasi K.K., Mabry J.M., Mckinley G.H., Cohen R.E. (2010). Relationships between Water Wettability and Ice Adhesion. ACS Appl. Mater. Interfaces.

[26] Nowak A.P., Gross A.F., Sherman E., Seebergh J.E., Dalby G.R., Berry D.H. (2015). Coatings, Coating Compositions, and Methods For Delaying Ice Formation. US Patent.

[27] Tuteja A., Kota A.K., Kwon G., Mabry J.M. (2011). Superhydrophilic and oleophobic porous materials and methods for making and using the same. US Patent.

[28] Zhang H., Lamb R.N., Jones A.W. (2004). Durable superhydrophobic coating. US Patent.

[29] Onda T., Shibuichi S., Satoh N., Tsujii K. (1996). Super-Water-Repellent Fractal Surfaces. Langmuir.

[30] Varanasi K.K., Deng T., Smith J.D., Hsu M., Bhate N. (2010). Frost formation and ice adhesion on superhydrophobic surfaces. Appl. Phys. Lett..

[31] Kulinich S.A., Farzaneh M. (2009). How wetting hysteresis influences ice adhesion strength on superhydrophobic surfaces. Langmuir.

[32] Menini R., Ghalmi Z., Farzaneh M. (2011). Highly resistant icephobic coatings on aluminum alloys. Cold Reg. Sci. Technol..

[33] Farhadi S., Farzaneh M., Kulinich S.A. (2011). Anti-icing performance of superhydrophobic surfaces. Appl. Surf. Sci..

[34] Tourkine P., Le Merrer M., Quéré D. (2009). Delayed Freezing on Water Repellent Materials. Langmuir.

[35] Wang F., Li C., Lv Y., Lv F., Du Y. (2010). Ice accretion on superhydrophobic aluminum surfaces under low-temperature conditions. Cold Reg. Sci. Technol..

[36] Kozera R., Przybyszewski B., Żołyńska K., Boczkowska A., Sztorch B., Przekop R.E. (2020). Hybrid modification of unsaturated polyester resins to obtain hydro-and icephobic properties. Processes.

[37] Varanasi K.K., Deng T., Hsu M.F., Bhate N. Design of Superhydrophobic Surfaces for Optimum Roll-Off and Droplet Impact Resistance. Proceedings of the ASME 2008 International Mechanical Engineering Congress and Exposition.

[38] Khaskhoussi A., Calabrese L., Patané S., Proverbio E. (2021). Effect of Chemical Surface Texturing on the Superhydrophobic Behavior of Micro–Nano-Roughened AA6082 Surfaces. Materials.

[39] Król P., Król B. (2012). Surface free energy of polyurethane coatings with improved hydrophobicity. Colloid Polym. Sci..

[40] Liu M., Wang S., Jiang L. (2013). Bioinspired multiscale surfaces with special wettability. MRS Bull..

[41] Herminghaus S. (2000). Roughness-induced non-wetting. Eur. Lett..

[42] Feng L., Zhang Y., Xi J., Zhu Y., Wang N., Xia F., Jiang L. (2008). Petal effect: A superhydrophobic state with high adhesive force. Langmuir.

[43] Miwa M., Nakajima A., Fujishima A., Hashimoto K., Watanabe T. (2000). Effects of the Surface Roughness on Sliding Angles of Water Droplets on Superhydrophobic Surfaces. Langmuir.

[44] Hirt C.W., Nichols B.D. (1981). Volume of fluid (VOF) method for the dynamics of free boundaries. J. Comput. Phys..

[45] Alla H., Freifer S., Roques-Carmes T. (2011). A computational fluid dynamics model using the volume of fluid method for describing the dynamics of spreading of Newtonian fluids. Colloids Surf. A Physicochem. Eng. Asp..

[46] Arzpeyma A., Bhaseen S., Dolatabadi A., Wood-Adams P. (2008). A coupled electro-hydrodynamic numerical modeling of droplet actuation by electrowetting. Colloids Surf. A Physicochem. Eng. Asp..

[47] El Ganaoui M., Mohammed B., Alla H. (2006). Numerical Investigation of a Drop/Surface Interaction.

[48] Nichita B.A., Zun I., Thome J.R. A VOF method coupled with a dynamic contact angle model for simulation of two-phase flows with partial wetting. Proceedings of the 7th International Conference on Multiphase Flow, ICMF 2010.

[49] Gunjal P.R., Ranade V.V., Chaudhari R.V. (2003). Experimental and computational study of liquid drop over flat and spherical surfaces. Catal. Today.

[50] Gunjal P.R., Ranade V.V., Chaudhari R. (2005). V Dynamics of drop impact on solid surface: Experiments and VOF simulations. AIChE J..

[51] Liu C., Liu Q., Jin R., Lin Z., Qiu H., Xu Y. (2020). Mechanism analysis and durability evaluation of anti-icing property of superhydrophobic surface. Int. J. Heat Mass Transf..

[52] Chamakos N.T., Kavousanakis M.E., Boudouvis A.G., Papathanasiou A.G. (2016). Droplet spreading on rough surfaces: Tackling the contact line boundary condition. Phys. Fluids.

[53] Pilch-Pitera B. (2014). Polyurethane powder coatings containing polysiloxane. Prog. Org. Coat..

[54] Seyfi J., Jafari S.H., Khonakdar H.A., Sadeghi G.M.M., Zohuri G., Hejazi I., Simon F. (2015). Fabrication of robust and thermally stable superhydrophobic nanocomposite coatings based on thermoplastic polyurethane and silica nanoparticles. Appl. Surf. Sci..

[55] Stalder A.F., Kulik G., Sage D., Barbieri L., Hoffmann P. (2006). A snake-based approach to accurate determination of both contact points and contact angles. Colloids Surf. A Physicochem. Eng. Asp..

